# Liver microRNA hsa-miR-125a-5p in HBV Chronic Infection: Correlation with HBV Replication and Disease Progression

**DOI:** 10.1371/journal.pone.0065336

**Published:** 2013-07-03

**Authors:** Nicola Coppola, Nicoletta Potenza, Mariantonietta Pisaturo, Nicola Mosca, Gilda Tonziello, Giuseppe Signoriello, Vincenzo Messina, Caterina Sagnelli, Aniello Russo, Evangelista Sagnelli

**Affiliations:** 1 Department of Mental Health and Public Medicine, Section of Infectious Diseases, Second University of Naples, Naples, Italy; 2 Department of Life Sciences, Second University of Naples, Caserta, Italy; 3 Department of Mental Health and Public Medicine, Section of Statistics, Second University of Naples, Naples, Italy; 4 Division of Infectious and Tropical Diseases, AORN Sant'Anna e San Sebastiano di Caserta, Caserta, Italy; 5 Department of Clinical and Experimental Medicine and Surgery "F. Magrassi e A. Lanzara", Second University of Naples, Naples, Italy; Drexel University College of Medicine, United States of America

## Abstract

To study in HBsAg chronic carriers the expression of liver hsa-miR-125a-5p and its correlation with liver HBV-DNA values and clinical presentation, 27 consecutive Caucasian, HBsAg/anti-HBe/HBV-DNA-positive patients who were naive to nucleos(t)ide analogues and interferon therapy and had no marker of HCV, HDV or HIV infection and no history of alcohol intake were enrolled. For each patient, liver HBV DNA and liver hsa-miR-125a-5p were quantified by real-time PCR in relation to β-globin DNA or RNU6B, respectively. Liver fibrosis and necroinflammation were graded by applying Ishak's scoring system. Liver hsa-miR-125a-5p was detected in all patients enrolled and a correlation between its concentration and liver HBV DNA was demonstrated (p<0.0001). Higher liver hsa-miR-125a-5p concentrations were observed in patients with HBV-DNA plasma level >10^3^ IU/ml (p<0.02), in those with HAI >6 (p = 0.02) and those with fibrosis score >2 (p<0.02) than in patients with lower scores. Higher HBV-DNA liver concentrations were found in patients with abnormal AST (p = 0.005) and ALT serum levels (p = 0.05), in those with serum HBV DNA higher than 10E3 IU/mL (p = 0.001) and those with fibrosis score >2 (p = 0.02) than in patients with a lower load. By multivariate logistic regression analysis, liver hsa-miR-125a-5p was identified as an independent predictor of disease progression: O.R. = 4.21, C.I. 95%  = 1.08–16.43, p<0.05, for HAI >6; O.R. = 3.12, C.I. 95%  = 1.17–8.27, p<0.05, for fibrosis score >2. In conclusion, in HBsAg/anti-HBe-positive patients, the liver hsa-miR-125a-5p level correlated with liver and plasma HBV-DNA values and was associated to a more severe disease progression.

## Introduction

Hepatitis B Virus (HBV) affects over 350 million people worldwide and is one of the leading causes of cirrhosis and hepatocellular carcinoma (HCC) [1]–[5]. The severity of chronic hepatitis B (CHB) is variable, with a clinical presentation ranging from a healthy HBV carriage to the more severe expressions of the disease, and with a clinical course of the illness ranging from a benign, indolent progression over decades to a rapid evolution to liver cirrhosis and HCC [6]–[9]. Several factors have been linked to the severity of CHB: virus-related factors (genotype, viral strain, viral load, length of infection), host-related factors (age at the time of infection, immune response to HBV), co-morbidities (immunosuppression of varying nature, coinfection with hepatitis delta virus, human immunodeficiency virus, hepatitis C virus) or environmental factors (alcohol abuse) [10]–[19].

MicroRNAs (miRNAs) are small non-coding RNAs that modulate gene expression at a post-transcriptional level by inhibiting translation of complementary mRNAs and/or targeting them for degradation [20], [21]. MicroRNAs play crucial roles in embryo development, maintenance of stem cell character, cell differentiation and apoptosis [22]–[24]. In addition, recent studies indicate that miRNAs are important regulators of virus-host interactions [25], [26]. With regard to HBV, it has recently been shown that hsa-miR-125a-5p, a microRNA expressed in the human liver, is able to target a viral sequence within the overlapping polymerase and surface antigen coding regions [27]. This interaction was demonstrated with a validation test based on cultured PLC/PRF/5, a cell line harboring copies of HBV genome and secreting the surface antigen (HBsAg) in the culture medium. In this system, transfection of an hsa-miR-125a-5p-mimic induced a marked decrease in the expression of HBsAg, whereas the transfection of a miRNA inhibitor increased the expression of HBsAg. The viral sequence targeted by hsa-miR-125a-5p encodes amino acids falling within a segment of the extracellular pre-S1 domain of HBsAg that is essential for cell binding. This may pose constraints for the virus not to mutate in this region. To this regard, a BLAST search of nucleotide collections at NCBI, using as a query the newly-identified miRNA-pairing sequence of HBV, revealed that over 4200 identical HBV sequences had been deposited, belonging to various genotypes, subtypes, serotypes or isolates [26]. Very recently, the effect of hsa-miR-125a-5p on the HBV gene expression was confirmed by an independent study [28]. In the HepG2.2.15 cell model of hepatitis B virus replication, the analysis of a panel of 814 miRNAs revealed that iron treatment, which increased HBV replication, resulted in a decreased hsa-miR-125a-5p expression, whereas TGF-β treatment, which decreased HBV replication, increased hsa-miR-125a-5p expression [28]. Taken together, these data indicate that hsa-miR-125a-5p is able to suppress HBV replication in cultured hepatocytes.

The present study investigates the hepatic expression of hsa-miR-125a-5p, its possible correlation with liver HBV DNA and the entity of liver damage in 27 patients with HBsAg/anti-HBe/HBV-DNA-positive biopsy-proven CHB.

## Patients and Methods

### Patients

The patients were selected at two Liver Units in southern Italy, one in Naples and one in Caserta, both associated to the School of Medicine of the Second University of Naples, Italy, and cooperating in several investigations with the same clinical approach and the same laboratory methods [29], [30].

Twenty-seven consecutive HBsAg/anti-HBe/HBV-DNA-positive Caucasian patients who were naive to nucleos(t)ide analogues and interferon therapy and who had no marker of HCV, HDV or HIV infection, no history of alcohol intake and no clinical, biochemical or US signs of liver cirrhosis were included in this investigation. These 27 patients underwent a diagnostic liver biopsy at one of the two above-mentioned clinical centers from April 2007 to March 2011.

In each case, liver biopsy was advised by the physicians in care, informed consent was signed by the patient and a liver specimen of at least 2.5 cm in length was always obtained. Two fragments of 1.5 mm in length, usually not informative for a histological diagnosis, were cut away from the two extremities of the liver biopsy and stored at −80°C in RNAlater solution (Qiagen GmbH, Hilden, Germany) for subsequent molecular analyses. For each patient, a plasma sample was collected on the same day that the liver biopsy was performed and stored at −80°C. Samples were collected for diagnostic purposes as part of routine care by the attending physicians.

### Ethics Statement

All patients provided written, informed consent for the collection and storage of biological samples and for the anonymous use of their data in clinical research, in accordance with the guidance set out by the Ethics Committee of the Azienda Ospedaliera of the Second University of Naples. Approval for the specific study was not required when the study began in April 2007. All procedures used in the study were in accordance with the current international guidelines, with the standards on human experimentation of the Ethics Committee of the Azienda Ospedaliera of the Second University of Naples, Italy, and with the Helsinki Declaration of 1975, revised in 1983.

### Liver histology

The liver specimens, in each case more than 2 cm in length, were fixed in 10% neutral buffered formalin, embedded in paraffin and stained with hematoxylin and eosin and Masson's trichrome method. Liver biopsies were examined by a pathologist who was unaware of the virological and clinical data. Necroinflammatory lesions (histological activity index, HAI) and fibrosis were assessed according to the Ishak scoring system [31].

### Molecular Biology Techniques

The two small pieces of liver tissue cut away from the liver biopsies were homogenized by TissueLyser (Qiagen GmbH, Hilden, Germany) for 30 seconds at 30 Hz.; the DNA was then extracted using microspin columns (QIAamp DNA kit, Qiagen GmbH, Hilden, Germany). Plasma DNA was extracted from 200 ul of plasma using microspin columns (QIAamp DNA Blood kit, Qiagen GmbH, Hilden, Germany). The DNA extracted from plasma and liver homogenates was analyzed for the presence of the HBV genome by performing a real-time PCR with a wide range of linearity in a Light-cycler 1.5 (Roche Diagnostics, Branchburg, NJ, USA) [32]. An external standard curve was made to quantify the HBV genomes present in the samples; the standard was a PCR product cloned with the TA cloning system (Invitrogen k2000-01, Invitrogen, Carlsbad, CA, USA), and 8 uL of the appropriately diluted plasmid was used to generate the standard curve. HBV DNA in the liver tissue was quantified in relation to â-globin DNA (LightCycler Control kit Fast star DNA Master Hyprobe, Roche Diagnostics, Branchburg, NJ, USA), a DNA present in all human cells and thus used as a positive control, using LightCycler quantification software (Roche Diagnostics, Branchburg, NJ, USA). The results were expressed as a number of IU/hepatic cell. The liver HBV DNA and â-globin DNA were evaluated by testing in the same run 2 replicates of the liver tissues of all 27 patients enrolled, and expressed as the mean of the two values observed.

Hepatitis B virus genotypes were determined by phylogenetic analysis of sequences of 400 nt of the S region, as previously described [33].

Liver hsa-miR-125a-5p was detected and quantified by real-time PCR. Total RNA was extracted by mirVana^TM^ miRNA isolation kit (Ambion) from liver tissues homogenized by TissueLyser, as described above, and the RNA concentration was determined spectrophotometrically (NanoDrop 2000c, ThermoScientific). RT-PCR tests for hsa-miR-125a-5p and RNU6B (used as a reference gene) were carried out using TaqMan® miRNA assays from Applied Biosystems according to the manufacturer's protocol. The expression level of hsa-miR-125a-5p in each liver sample was normalized to RNU6B by using the 2^−DCt^ method and reported as arbitrary units (AU) [34]. The data are mean ± SD of three replicates.

### Routine analysis

HBV and HDV serum markers were sought using commercial immunoenzymatic assays (Abbott Laboratories, North Chicago, IL, USA, for HBsAg, anti-HBs and anti-HBc, and DiaSorin, Saluggia, VC, Italy, for HBeAg, anti-HBe and anti-HDV). The anti-HCV antibody was sought using a 3^rd^ generation commercial immunoenzymatic assay (Ortho Diagnostic Systems, Neckargemund, Germany). Antibodies to HIV 1 and 2 were sought using a commercial ELISA (Abbott Lab., North Chicago, Ill, USA). Liver function tests were performed by routine methods.

### Statistical Analysis

Continuous variables were summarized as mean and standard deviation, and categorical variables as absolute and relative frequencies. The differences were evaluated by Wilcoxon signed ranks test, categorical variables were compared by chi-square test, using exact procedures if needed. Odds ratios, with 95% confidence intervals (CI), were estimated by a logistic regression model for evaluating the relationship between age, liver hsa-miR125a-5p, liver HBV DNA and HAI and fibrosis. A p value <0.05 was considered to be statistically significant.

## Results

The demographic, biochemical, virological, histological and biomolecular data obtained at enrolment are shown in Table 1. Seventeen (63%) patients were males and the median age was 41 years (range 22–61). Surgery was the most frequent risk factor for the acquisition of HBV infection (59.3%), followed by unsafe sexual intercourse (14.8%) and by intrafamily exposure to an HBV chronic carrier (11.1%). Serum aminotransferases (aspartate-aminotransferase, AST, and alanine-aminotransferase, ALT) were normal in 17 patients (62.9%) and abnormal in 10 (37.1%); the mean (±SD) HAI score was 4.74 (±2.45), and the mean fibrosis score was 1.3 (±1.1), single scores ranging from 0 to 3 (Table 1). Both plasma and liver HBV-DNA were detected in all 27 patients; the mean plasma HBV-DNA level was 4×10^6^±7.92×10^6^ IU/ml and that of liver HBV DNA was 1.66±4.81 IU/cell (median value 0.088 IU/cell). In all cases HBV genotype D, the most common in Italy, was detected.

**Table 1:** Initial demographic, virological and clinical characteristics of the 27 HBsAg-positive patients.

Patient#	Age(years)	Sex	Risk factors	ALT (x n. v.)	Serum HBV DNA (IU/mL)	HAIScore	Fibrosisscore	Liver hsa-miR-125a-5p AU (Mean±SD)	Liver HBV DNA(IU/cells) (Mean±SD))
**# 1**	43	M	None	0.6	2.1E3	0	0	0.06±0.037	0.0092±0.0014
**#2**	40	M	Sex	1.0	4.7E3	4	0	0.95±0.014	0.0115±0.0080
**#3**	35	M	Surgery	2	1.7E5	6	1	1.43±0.011	0.0243±0.0188
**#4**	33	M	Surgery	1.0	1.20E2	3	1	2.62±0.539	0.0028±0.00007
**# 5**	45	M	Sex	1.0	2E1	3	1	2.03±0.092	0.7162±0.0110
**#6**	56	M	None	4.9	4.60E4	6	1	0.56±0.019	0.1280±0,0010
**#7**	53	M	Surgery	0.6	4.1E3	5	1	2.46±0.077	0.0062±0.0002
**#8**	35	M	Transfusion	2.6	5.1E6	7	3	3.49±0.213	0.1859±0.0800
**#9**	40	M	None	4.2	3.40E7	6	3	2.81±0.186	0.1565±0.0313
**#10**	50	M	Surgery	10.1	9.1E6	9	3	5.66±0.477	0.4257±0.0999
**#11**	33	M	Surgery	1.24	3.68E2	7	3	2.37±0.483	0.0005±0.0003
**#12**	34	M	Familiarity	6.5	1.1E6	8	3	2.62±0.416	6.8395±1.5888
**#13**	46	M	Surgery	1.0	4.80E4	9	3	4.66±0.102	0.3046±0.0577
**#14**	55	F	Sex	0.2	3.20E1	6	2	1.17±0.421	0.0380±0.0265
**#15**	61	M	Surgery	0.9	3.30E5	7	2	1.48±0.089	0.0385±0.0225
**#16**	41	F	Surgery	2	1.10E6	8	2	0.6±0.148	18.5937±5.0527
**#17**	54	F	Surgery	0.5	6.80E1	3	1	0.74±0.057	0.0436±0.0134
**#18**	22	F	Surgery	0.4	7.20E3	3	1	0.92±0.068	0.0929±0.0073
**#19**	23	F	Familiarity	1.9	2.60E4	4	0	3.04±0.521	16.8269±2.711
**#20**	40	M	Familiarity	0.8	1.20E4	1	3	0.96±0.048	0.0984±0.034
**#21**	35	F	Familiarity	0.5	3.14E3	3	1	0.78±0.046	0.1358±0.0363
**#22**	55	F	Surgery	1.5	5.1E4	5	1	1.22±0.019	0.0881±0.0054
**#23**	44	F	Surgery	0.5	3.5E3	3	0	0.80±0.200	0.0146±0.0134
**#24**	34	F	Surgery	0.4	1.40E2	1	0	1.07±0.093	0.0267±0.0096
**#25**	40	M	Surgery	1.0	1.10E5	5	0	0.37±0.021	0.0083±0.0015
**#26**	52	M	Surgery	0.5	5.1E3	2	0	0.93±0.120	0.0941±0.0326
**#27**	51	F	Surgery	0.5	4.70E3	4	0	0.77±0.057	0.0060±0.0053

**Footnotes**: F = Female; M = Male, None =  no risk factor; HAI: Histological Activity Index; n.v. = normal values.

**Figure 1:**
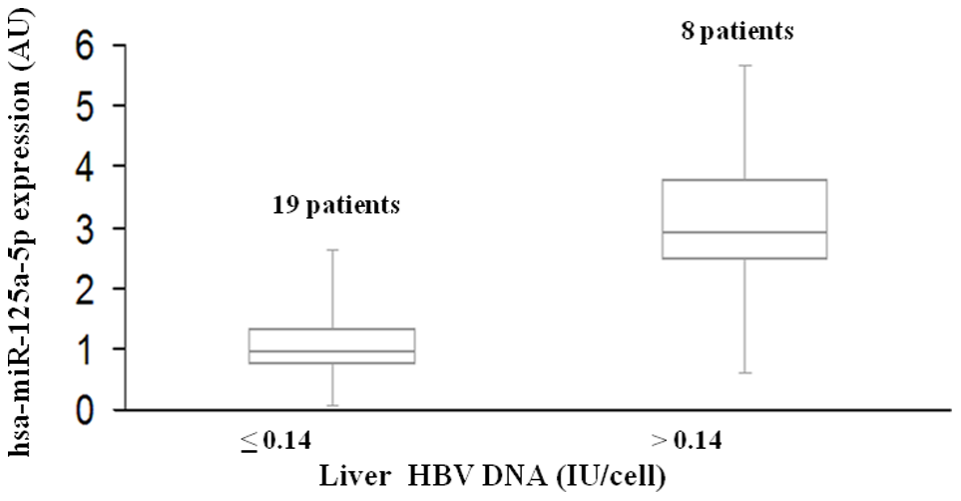
Correlation of hsa-miR-125a-5p expression with the levels of liver HBV DNA. Box plots of hsa-miR-125a-5p expression in two groups of patients with lower (≤0.14) or higher (>0.14) levels of liver HBV DNA. The vertical lines indicate the value ranges, the horizontal boundaries of the boxes represent the first and third quartile; n indicates the number of cases. The p value for the comparison of the two data sets was <0.0001 (Wilcoxon test).

Liver hsa-miR-125a-5p was detected in all patients enrolled, with a mean concentration of 1.72±1.54 AU (median value 1.17AU). There was a correlation between the expression levels of liver hsa-miR-125a-5p and those of liver HBV DNA. In fact, using a 0.14 cut-off value, patients were divided into two subgroups with very different levels of hsa-miR-125a-5p, the 19 patients with liver HBV DNA ≤0.14 IU/cell (median and range: 0.95AU, 0.06–2.62) and the 8 with HBV DNA >0.14 IU/cell (2.93 AU, 0.60–5.66, p<0.0001) (Figure 1).

The levels of liver HBV DNA and those of liver hsa-miR-125a-5p were also analyzed according to the patients' sex, age, AST and ALT serum values, HBV-DNA plasma level and HAI and fibrosis scores. In a univariate analysis, no association was found between the hsa-miR-125a-5p liver concentration and sex, age or aminotransferase serum levels, whereas significantly higher hsa-miR-125a-5p liver concentrations were observed in patients with HBV-DNA plasma levels >10^3^ IU/ml [median 1.48 (0.37–5.66) vs 0.94 (0.06–2.62), p = 0.02], in those with HAI >6 [2.62 (range 0.60–5.66) vs 0.95 (0.06–3.04), p = 0.02] and in those with fibrosis score >2 [2.81 (0.96–5.66) vs 0.94 (0.06–3.04), p = 0.02] (Table 2). As regards the HBV-DNA liver concentration, no correlation was observed with age, sex or HAI (Table 2), whereas significantly higher HBV-DNA liver concentrations were found in patients with abnormal AST serum levels [0.19 (0.02–18.59) vs 0.03 (0.0005–0.71), p = 0.002], in those with abnormal ALT serum levels [0.17 (0.0005–18.59) vs 0.032 (0.0005–0.72, p<0.05], those with serum HBV DNA higher than 10^3^ IU/mL [0.17 (0.00052–18.59) vs 0.038 (0.003–0.72), p<0.001], and those with a fibrosis score higher than 2 [0.186 (0.0005–6.84) vs 0.038 (0.003–18.59), p<0.02] (Table 2).

**Table 2:** Liver hsa-miR-125a-5p and liver HBV-DNA concentration according to demographic, initial biochemical, virological and histological data.

		Liver hsa-miR-125a-5p, AU median (range)		Liver HBV DNA, IU/cell median (range)	
Parameter	Number		* *p-value*		* *p-value*
**Sex:**					
**Male**	17	2.03 (0.37–5.66)		0.094 (0.00052–6.8395)	0.786
**Female**	10	0.86 (0.60–3.04)	0.083	0.066 (0.00605–18.594)	
**Age:**					
**≤40 years**	13	1.43 (0.37–3.49)	0.131	0.093 (0.0005280–16.8269)	0.940
**>40 years**	14	1.05 (0.06–5.66)		0.066 (0.00605–18.59375)	
**AST:**					
**≤1.0×n.v.**	18	0.95 (0.06–4.66)	0.095	0.0324 (0.0005280–0.7162)	0.002
**>1.0×n.v.**	9	2.62 (0.56–5.66)		0.1859 (0.02435–18.59375)	
**ALT:**					
**≤ 1.0×n.v.**	17	0.95 (0.06–4.66)	0.059	0.032 (0.0005280–0.7162)	0.027
**> 1.0×n.v.**	10	2.50 (0.56–5.66)		0.1712 (0.0005280–18.594)	
**P HBV DNA:**					
**≤10E3 IU/ml**	14	0.94 (0.06–2.62)	0.019	0.038 (0.00285–0.7162)	0.001
**>10E3 IU/ml**	13	1.48 (0.37–5.66)		0.171 (0.0005280–18.594)	
**HAI score:**					
**≤6**	20	0.95 (0.06–3.04)	0.019	0.040825 (0.00285–16.83)	0.072
**>6**	7	2.62 (0.60–5.66)		0.3046 (0.0005280–18.594)	
**Fibrosis score:**					
**≤2**	20	0.94 (0.06–3.04)	0.013	0.0383 (0.00285 –18.594)	0.019
**>2**	7	2.81 (0.96–5.66)		0.1859 (0.0005280–6.,8395)	

* Wilcoxon rank test. Footnotes: P-HBV DNA =  Plasma HBV DNA; HAI =  Histological Activity Index; n.v. = normal values.

To confirm the association between liver hsa-miR-125a-5p and the histological lesions (HAI and fibrosis) and to avoid a possible confounding effect of other factors such as age and the liver HBV-DNA level, a multivariate logistic regression analysis was performed. Multivariate analysis identified liver hsa-miR-125a-5p as an independent predictor of HAI >6 (O.R.: 4.21, C.I. 95%: 1.08–16.43, p<0.05) and of a fibrosis score >2 (O.R.: 3.12, C.I. 95%: 1.17–8.27, p<0.05) (Table 3).

**Table 3:** Logistic regression analysis for independent predictors of HAI >6 and fibrosis score >2 (Ishak).

Parameters	Odd Ratios	C.I. 95%		*p- values*
		Lower	Upper	
**HAI >6**				
**Age**	1.093	0.940	1.271	0.246
**Liver hsa-miR-125a-5p**	4.214	1.080	16.435	0.038
**Liver HBV DNA**	1.204	0.963	1.506	0.104
**Fibrosis score >2**				
**Age**	0.986	0.877	1.109	0.818
**Liver hsa-miR-125a-5p**	3.116	1.174	8.272	0.023
**Liver HBV DNA**	1.103	0.897	1.356	0.353

**Figure 2:**
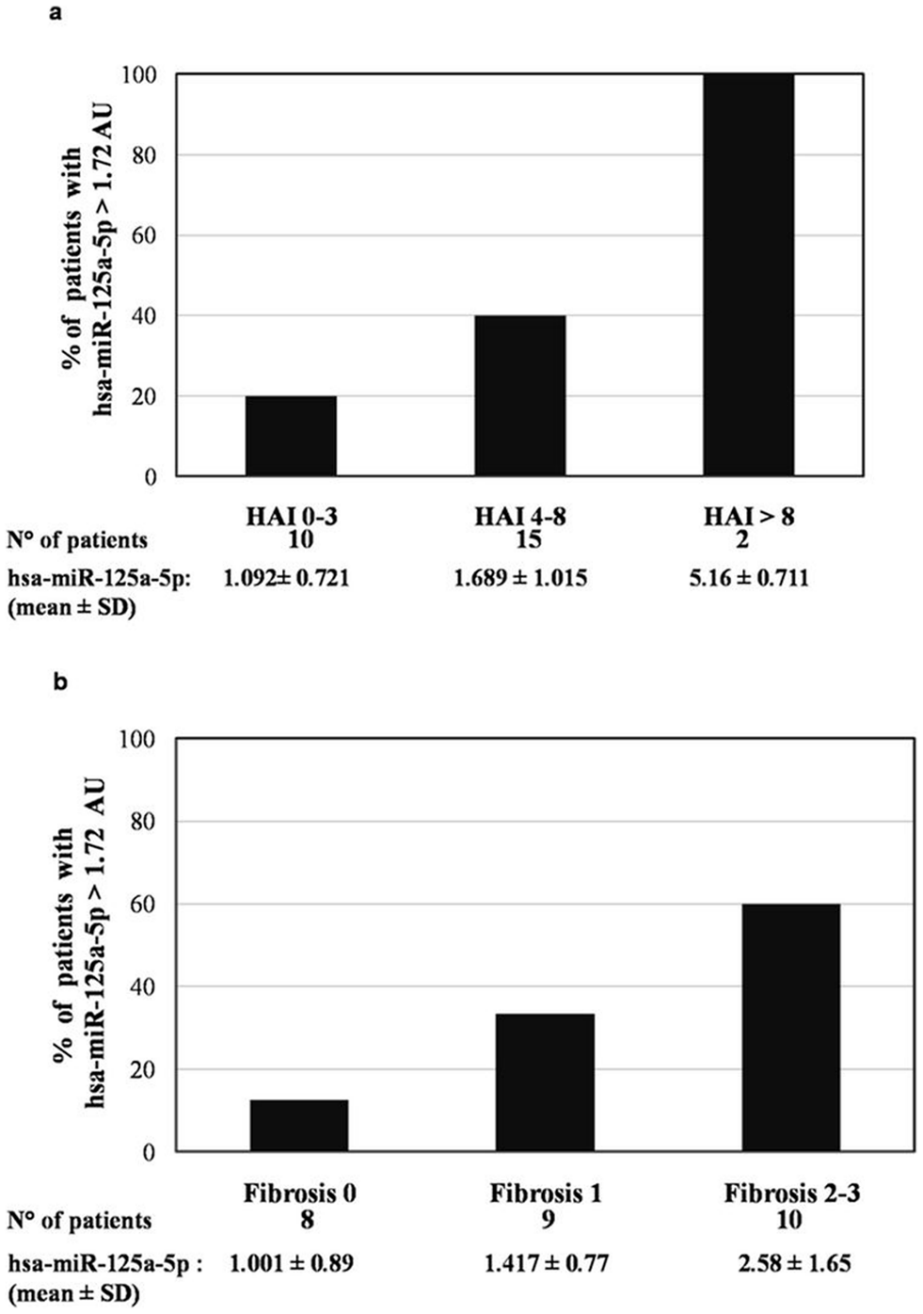
hsa-miR-125a-5p according to the HAI score (a) and fibrosis score (b).

**Figure 3:**
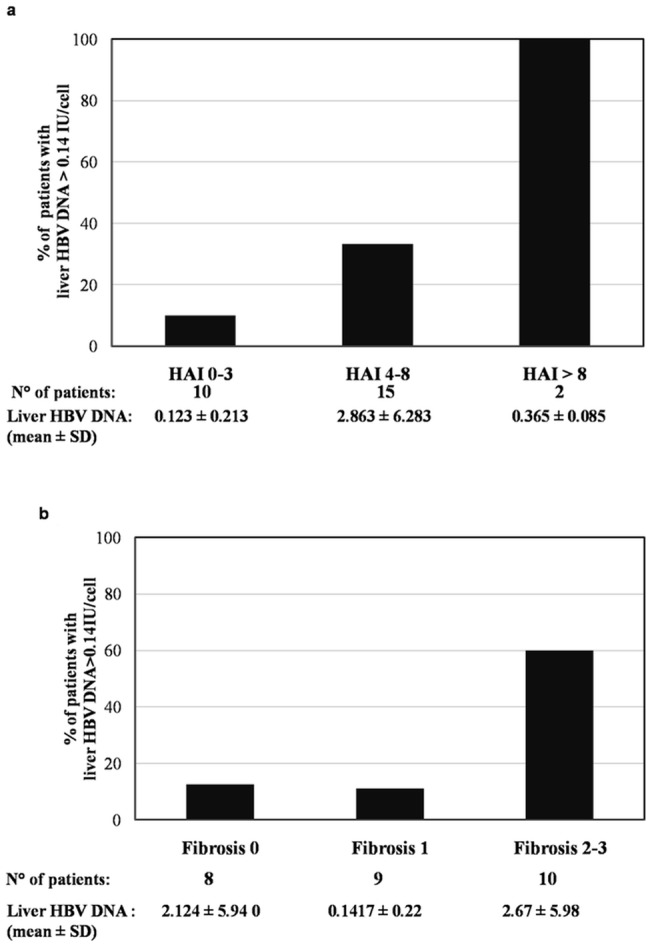
Liver HBV DNA according to the HAI score (a) and fibrosis score (b).

In addition, a progressive increase in the mean value of liver hsa-miR-125a-5p paralleled the increase in the HAI scores (Figure 2a) and in the fibrosis scores (Figure 2b). Similarly, there was a progressive increase in the prevalence of patients with a liver HBV-DNA value >0.14 IU/cell and in patients with liver hsa-miR-125a-5p >1.72 AU, which reflected the increase in the HAI scores (Figure 3a) and fibrosis scores (Figure 3b).

## Discussion

Many cellular mechanisms, such as innate immunity and cell signaling pathways contribute to the control of viral pathogenesis. Recently, some studies have highlighted cellular or viral miRNAs as a new class of regulators in viral pathogenesis [26]. HBV is a widespread human pathogen and chronic HBV infection is a major risk factor for HCC [1]–[5]. Some miRNAs have been shown to affect HBV gene expression and replication in cultured cells by direct binding to the viral transcripts. They include hsa-miR-210, hsa-miR-199-3p [35], hsa-miR-125a-5p [27], [28] and hsa-miR-151-5p [28]. However, their activity in HBV-infected tissues awaits further studies to confirm their correlation to HBV infection *in vivo*, to explore their value as possible biomarkers for disease progression and, possibly, to define new strategies for anti-HBV intervention.

We focused our attention on hsa-miR-125a-5p because of its anti-HBV activity established by independent studies [27], [28], and for evidence suggesting that its expression *in vitro* may be correlated to HBV exposure. To this regard, it has been reported that hsa-miR-125a-5p is expressed in the HBV-producing cell line HepG2.2.15 at levels at least 3-fold higher than in its parent cell line HepG2 [36]; ectopic expression of the HBV X protein in HepG2 cells increases the expression level of hsa-miR-125a-5p [37]. Finally, the target site for this microRNA is well conserved in the viral population [26], supporting a significant role in HBV-host interaction.

In this study, patients with HBsAg/anti-HBe-positive chronic hepatitis were enrolled and the expression levels of liver hsa-miR-125a-5p miRNA were determined, along with several bioclinical. and histological parameters. The hsa-miR-125a-5p miRNA was found to be expressed in the liver of all patients, and its levels correlated with HBV DNA concentrations in both the liver and plasma. The approximately three-fold increase in the expression of liver hsa-miR-125a-5p in patients with HBV-DNA levels higher than 0.14 IU/cell provides strong evidence that liver exposure to HBV induces the expression of hsa-miR-125a-5p, as suggested by previous *in-vitro* studies [36], [37]. This finding, together with the knowledge that hsa-miR-125a-5p can suppress HBV replication in cell cultures [27], [28], suggests that this miRNA may be part of a negative feedback limiting HBV replication. In fact, transfection of an hsa-miR-125a-5p-mimic in cultured PLC/PRF/5 induced a marked decrease in the expression of HBsAg, whereas transfection of a miRNA inhibitor increased the expression of HBsAg [27]. This effect would be beneficial for the host but also for the virus, which, co-opting a cellular miRNA to self-limit its own replication, would establish a persistent infection. Similar results were obtained studying the effects of cellular miRNAs on HIV-1 replication. In 2007 Huang et al. showed that human microRNAs miR-28, miR-125b, miR-150, miR-223 and miR-382 are over-expressed in resting CD4^+^ T cells and are able to target the 3′ end of the human HIV-1 RNA, thus silencing almost all viral messengers. Specific inhibitors of these cellular miRNAs reversed their effects, measured either as HIV-1 translation or HIV-1 virus production, thereby arguing for a role of these cellular miRNAs in HIV latency [38].

The present study also identified liver hsa-miR-125a-5p as an independent predictor of moderate and severe stages of HBV disease, since high expression levels of liver hsa-miR-125a-5p were found to be associated with HAI scores >6 and fibrosis scores >2. It may be hypothesized that this association is the expression of the negative feedback of has-miR-125a-5p on HBV replication, as high liver hsa-miR-125a-5p levels are induced by high levels of HBV DNA, which in turn are associated to a more severe liver disease [12]. These data seem interesting despite the fact that the liver disease in our patients was mild in most cases, with abnormal ALT serum values only in 37.1% and higher HAI and fibrosis scores only in 25.9%. Thus, although the number of patients with a severe liver disease was low, a clear correlation between high hsa-miR-125a-5p levels and the severity of the liver disease was observed.

In conclusion, this study showed that in a group of HBsAg/anti-HBe-positive patients the expression of liver hsa-miR-125a-5p correlated with the HBV load and severity of the liver disease. These data provide the basis for further studies aimed at understanding the mechanism of HBV induction of liver hsa-miR-125a-5p, its measurements in plasma or PBMC for a possible definition of new non-invasive markers of HBV-associated liver diseases and possible development of new anti-HBV strategies based on the over-induction of hepatic hsa-miR-125a-5p.
